# Quitline referral vs. self-help manual for tobacco use cessation in the Emergency Department: a feasibility study

**DOI:** 10.1186/1471-227X-7-15

**Published:** 2007-09-14

**Authors:** Nicola EE Schiebel, Jon O Ebbert

**Affiliations:** 1 Department of Emergency Medicine, Mayo Clinic, Rochester, Minnesota, USA; 2 Department of Internal Medicine, Mayo Clinic, Rochester, Minnesota, USA

## Abstract

**Background:**

Tobacco use counseling interventions delivered in the primary care setting are efficacious, but limited evidence exists regarding their feasibility or efficacy in the Emergency Department (ED). ED randomized controlled trials evaluating referral for outpatient tobacco use counseling have not had a single subject in the intervention groups attend scheduled clinic appointments. Telephone counseling potentially affords the opportunity to provide this population with individual counseling more conveniently than traditional clinic counseling. The purpose of this preliminary study was to evaluate the intervention completion rate among cigarette smokers enrolled through the ED in a tobacco quitline (QL) and to assess the feasibility of a randomized controlled trial assessing the efficacy of this intervention.

**Methods:**

We conducted a prospective, randomized, controlled, un-blinded pilot study enrolling cigarette smokers presenting to a tertiary-care ED. Patients indicating a desire to quit smoking were randomized to receive either proactive telephone counseling through a QL (intervention) or a self-help manual (control).

**Results:**

Of 212 smokers who indicated an interest in quitting, 20 subjects were randomized to the QL and 19 to control. Twenty-one did not meet inclusion criteria and 152 refused to participate. A total of 10 patients (50%) enrolled in the QL completed the full intervention. However, only a total of 20 patients (51%) were reached for follow-up at 3 or 6 months (10 in each arm). At 6-month follow-up a total of six subjects had either disconnected their phone, no longer lived at the provided phone number or had provided an incorrect number. Two declined to provide follow-up and the remainder could not be reached. Assuming all patients unavailable for follow-up were still smoking, the 7-day point prevalence smoking abstinence rate at 6 months was 20% (95% CI: 6 to 44%) for the QL group and 0% (95% CI: 0 to 15%) for the control group (p = 0.11).

**Conclusion:**

Compliance with the QL intervention was encouraging and may hold promise for providing needed tobacco use counseling to ED patients. Future studies are required, and should focus on more effective mechanisms to obtain outcome measures and a larger sample size.

**Trial Registration:**

NCT00394420

## Background

Cigarette smoking is the most preventable cause of death and disability in the United States and accounts for approximately 435,000 deaths annually. Available evidence suggests that tobacco use counseling interventions delivered in the primary care setting are practical and efficacious, but limited evidence exists regarding their feasibility or efficacy in the Emergency Department (ED) [1].

The ED is increasingly becoming the principal source of healthcare for patients unable to access primary care and preventive services. While the ED is recognized as a potential site to initiate preventive interventions such as smoking cessation counseling, resource constraints and low patient adherence pose significant barriers. Patients seen in the ED have high rates of cigarette smoking and many intend to quit [2]. A multicenter ED survey reported that 33% of smokers wanted an outpatient referral for counseling [3]. Prospective investigations of referral for counseling from the Emergency Department (ED), however, have demonstrated a lack of compliance with outpatient referral. A systematic review of tobacco use interventions in the ED [1] identified two randomized controlled trials evaluating referral from the ED for outpatient tobacco use counseling [4,5]. Not a single subject in the intervention groups attended or completed clinic appointments. The development of more practical tobacco use interventions associated with high patient adherence would potentially be a major step forward in public health.

Overall, the available evidence suggests a positive effect of proactive telephone counseling for increasing tobacco abstinence rates [6]. The U.S. Public Health Service clinical practice guideline found proactive telephone counseling to be effective (OR 1.2; 95% CI: 1.02–1.23) and recommended that it be used as a behavioral component in tobacco use interventions with a strength of evidence rating of "A" (i.e., "multiple well-designed randomized clinical trials, directly relevant to the recommendation, yielded a consistent pattern of findings") [7]. In addition, a meta- analysis of proactive telephone counseling using a best-evidence synthesis confirmed a significant increase in cessation rates compared with control conditions with pooled odds ratios of 1.34 (95% CI: 1.19–1.51) and 1.20 (95% CI: 1.06–1.37) at short- and long-term follow-up, respectively [8].

In a recent survey of patients presenting to the ED, 30% of current cigarette smokers were considering smoking cessation in the next month and 27% reported they would be interested in receiving telephone-based counseling for quitting tobacco use after the ED visit [9]. Telephone counseling affords the opportunity to provide individual counseling for tobacco use economically while allowing more flexibility and convenience for the patient compared to traditional clinic counseling [6]. Furthermore, the tobacco quitline (QL) provides a strategy whereby ED providers can capitalize on face-to-face interaction and engage patients in QL counseling that serves as "a behavioral extension to the clinician" [10]. However, a QL in conjunction with a tobacco use intervention in the ED have not been formally evaluated.

We undertook this pilot study to evaluate the intervention completion rate of ED patients who smoke cigarettes and are enrolled in proactive counseling through a QL. We used a *Fax-to-Quit *model which has been demonstrated to both feasible [11] and cost-effective [12] in the primary care setting. To date, this approach has not been evaluated in the ED setting. If adequate compliance with the intervention could be achieved, this pilot was also designed to assess the feasibility of conducting a randomized controlled trial to evaluate the efficacy of QL referral through the ED.

## Methods

### Study design

This pilot study was a prospective, randomized, controlled, un-blinded study using a convenience sample of cigarettes smokers recruited through the ED. Concealment of allocation was assured by remote randomization via a phone call to pharmacy after consent was obtained. The study protocol was approved by the Mayo Institutional Review Board prior to subject enrollment.

### Study setting & selection of participants

The study was conducted in the ED of a tertiary referral center in the Midwest. The ED is located in a community of 85,000 people with approximately 70,000 patient visits per year. The admission rate is 30%.

Subjects were enrolled between September 2003 and August 2004. We enrolled English speaking patients aged 18 or older who reported current daily cigarette smoking for at least one year and who indicated an interest in attempting to quit smoking.

Attending ED physicians and residents identified potential study participants by asking smokers if they were interested in participating in a research study that might help them quit. Recruitment was limited to times when study personnel were available to consent and enroll subjects. Study personnel were available approximately 50% of dayshifts, 20% of evenings and 5 % of night shifts.

Identified patients were then screened by study personnel to assess eligibility for the study. A templated interview form was utilized to ensure all inclusion/exclusion criteria were assessed. Patients were excluded if they were critically ill, in severe pain, presenting for psychiatric complaints, not available for telephone follow-up, admitted to the hospital, refused informed consent, or had no plans to quit. The final question presented to the prospective participants meeting all other criteria was "are you interested in stopping your smoking?" If they responded yes, the details of the study and the consent process were reviewed with them.

### Study protocol

Utilizing a computerized block randomization schedule, subjects were randomized in blocks of four to receive a proactive QL intervention through an established QL or a United States Public Health Service (USPHS) self-help manual. Proactive telephone counseling describes counseling that is initiated by a counselor rather than the subject. Both groups received strong advice to quit from study personnel.

Names and telephone numbers of patients randomized to the QL group were faxed to the QL, which then initiated contact with the patient. Multiple contact attempts were made over the following week by telephone. Patients in the QL were instructed to call the QL if they had not been reached in one week. QL counseling involved an initial 45-minute telephone session followed by up to four 10 to 15 minute follow-up sessions around their identified quit date. QL patients not successfully contacted within one week of enrollment were sent a letter inviting them to call, as well as information on strategies to help them quit.

### Measurements

Baseline demographic data, smoking history, discharge diagnosis, and scores on the Fagerström Test for Nicotine Dependence (FTND) and the Contemplation Ladder were obtained. Baseline data were collected by a study assistant.

The 6-item FTND is a validated scale with scores that range from 0 to 10, with higher scores indicating greater levels of nicotine dependence [13]. A score of 4 indicates a high probability of tobacco dependence.

The Contemplation Ladder is a measure of readiness to quit smoking [14]. It consists of an 11-point scale (range: 0 to 10) on which tobacco users rank their current level of motivation to quit. The Contemplation Ladder has been shown to be significantly associated with reported intention to quit, number of previous quit attempts, perceived co-worker encouragement, and socioeconomic status [14].

Both groups were scheduled for follow-up telephone calls to assess self-reported smoking status at 3 and 6 months from enrollment. Multiple attempts to reach subjects were made between the hours of 7 am to 7 pm on weekdays and 10 am to 4 pm on Saturdays. In order to avoid reporting bias, a separate staff of evaluators blinded to participant group allocation interviewed subjects.

### Data analysis

The primary outcome was completion of the QL intervention by the intervention group in order to assess the willingness of an ED population to participate in this type of counseling. Completion was defined as completing the baseline QL counseling and at least one telephone call around their quit date. Feasibility of a larger randomized controlled trial for efficacy of the intervention was also evaluated by recording completion rates for telephone outcome calls at 3 and 6 months after enrollment. Secondary outcome was the 7-day point prevalence smoking abstinence (i.e., no smoking in the last 7 days) at 3 and 6 months. Analysis was intention-to-treat and all patients unavailable for follow-up were assumed to be smoking. The sample size of 40 participants was determined by funding available for the pilot.

Data were summarized using medians with interquartile (IQR) or full ranges for continuous variables and percentages for categorical variables. For each percentage abstinence rate, the numerator and denominator are presented with 95% exact binomial confidence intervals. A 2-sided Fischer's exact test was used to compare abstinence rates between groups at 3- and 6-month follow-up. Discharge diagnoses were summarized as a crude measure of patient acuity.

## Results

**Figure 1:**
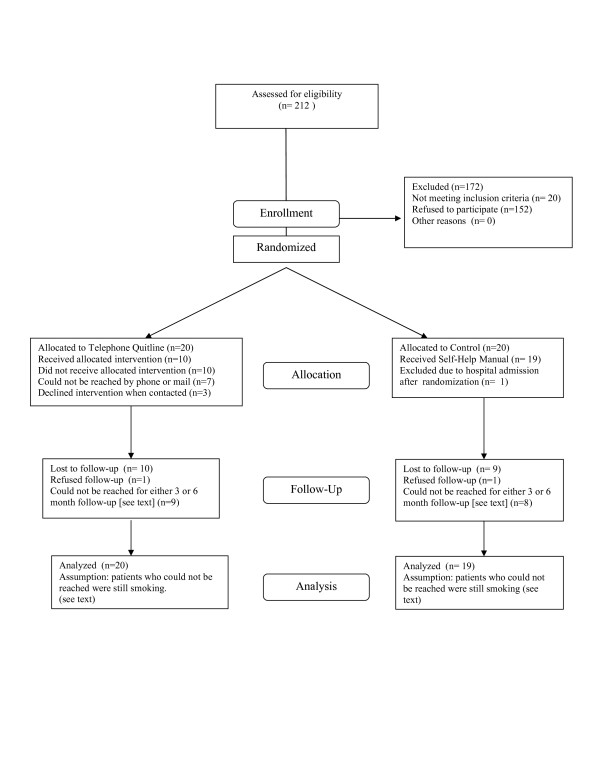
Study Flowchart.

Two hundred twelve smokers were screened by study personnel (Figure 1). Twenty did not meet inclusion criteria, including 7 who did not wish to discontinue smoking. One hundred fifty two met all other criteria, but refused to participate in research. Forty patients were enrolled and randomized. One patient was excluded after randomization because of inappropriate inclusion due to hospital admission. Of the remaining 39 patients, 20 subjects were assigned to the QL intervention and 19 to control. The groups were demographically and clinically comparable at baseline (Table 1) with the exception of a higher median number of cigarettes per day reported in the QL group compared to control (20 vs. 10).

**Table 1:** Baseline characteristics of Emergency Department patients in a pilot study of a tobacco use intervention linked to a tobacco quitline (N = 39)

Characteristic	Telephone Quitline, N = 20		Control, N = 19	
	No. (%)	Median (IQR)	No. (%)	Median (IQR)
Gender:				
Female	8 (40)		11 (58)	
Male	12 (60)		8 (42)	
Age		36 (24–42)		41 (29–44)
Caucasian	20 (100)		18 (95)	
Marital Status:				
Never	8 (40)		4 (21)	
Separated/divorced/widowed	3 (15)		5 (26)	
Married/living as married	9 (45)		10 (53)	
Highest education completed:				
Some high school	2 (10)		1 (5)	
High school graduate/GED	8 (40)		8 (42)	
Some college/technical school/vocational school	10 (50)		10 (52)	
Cigarrettes per day		20 (11–29)		10 (8–20)
Years smoked		15 (9–26)		18 (9–25)
Number of previous quit attempts:				
0	0		4 (21)	
1–2	9 (45)		8 (42)	
3–4	5 (25)		2 (11)	
5 or greater	6 (30)		5 (26)	
Contemplation ladder		8 (7–8)		7 (6–10)
FTND		5 (3–7)		4 (2–6)

IQR, interquartile range

Based on the discharge diagnoses, the overall acuity of illness in enrolled patients was low. The majority of patients had minor traumatic injuries or musculoskeletal complaints (15/20 QL; 8/19 control). Other diagnoses included minor upper respiratory illness, chest pain, headache, dysuria/urinary tract infection, dental pain, chemical exposure, deep venous thrombosis, renal colic and atrial fibrillation. Diagnoses did not differ by treatment group.

A total of 10 patients (50%) in the QL group completed the intervention. The median number of phone calls required to complete the first counseling session was 3 (range 2–6). Seven patients could not be reached by telephone or mail and 3 declined the intervention when contacted. The median number of calls made in attempts to reach this group was 6 (range 1–16). Baseline demographic and clinical characteristics of these two groups were similar, except for a trend towards a lower median FTND score in the group that completed the intervention (3.5; IQR 1, 6) versus the group that did not complete the QL (5.5; IQR 4, 7).

One QL subject declined both the intervention and any further contact for follow-up outcome telephone calls at the initial contact. Of the remaining 38 patients, only two patients (5%) could be reached for follow-up at both 3 and 6 months (both in QL). One reported smoking at both points and one had quit at both time points. Eighteen patients could be reached once at either the 3- or 6-month follow-up, resulting in a total of 20 (51%) contacted with 10 in each arm. One control group subject declined to provide any information when contacted for follow-up. The median number of calls made for patients who were eventually reached for follow-up was 3 (range 1–7) at 3 months and 1 (range 1–3) at 6-month follow-up. In contrast the median number of attempts for the patients we were unable to contact was 12 (range 1–29) at 3 months and 4.5 (range 1–21) at 6 months. At 6-month follow-up a total of six subjects had either disconnected their phone, no longer lived at the provided phone number, or had provided an incorrect number.

The 7-day point prevalence smoking abstinence rate at 3-month follow-up was 10% (2/20; 95% CI: 1 to 32%) for the QL group versus 5% (1/19, 95% CI: 0 to 26%) for the control group (p = 1.00). At 6 months, the QL group had a 20% abstinence rate (4/20; 95% CI: 6 to 44%) compared to 0% (0/19; 95% CI: 0 to15%) for the control group (p = 0.11).

## Discussion

This feasibility pilot trial of a smoking cessation intervention initiated through the ED provides important information regarding the success and failures of ED-based interventions. First, we observed that the QL intervention completion rate (50%) was considerably higher than previous ED studies where no subjects followed-up with outpatient appointments for tobacco use counseling [4,5]. Given the small numbers in this study, however, more research is needed to evaluate true participation rates for proactive QL interventions initiated in the ED. In addition, despite screening over 200 smokers, only 40 (20%) were enrolled in the study. Once enrolled, half were unreachable or became disinterested in any smoking cessation interventions. Overall, a system such as this appears to be time consuming, yet promising from the perspective of compliance with the intervention. Clearly, feasibility of a larger randomized controlled trial will first require a more efficient screening tool. Perhaps the most important issue is identifying patients who truly wish to cease smoking and referring them. Considerations such as targeting specific presenting complaints including chest pain or respiratory complaints may help increase interest in smoking cessation. Further research is required to assess whether this approach would improve efficiency of enrollment and lead to better success with follow-up.

Convenience may be an important factor affecting compliance in the ED population and the completion rate for phone counseling intervention in this small population is encouraging given the evidence already available supporting the efficacy of proactive QL counseling. Proactive QL counseling (i.e., telephone counselors initiate patient contact) has been shown to increase abstinence rates compared to a reactive QL (i.e., tobacco user initiates contact) in other populations [15], and proactive telephone counseling is recommended by the United States Public Health Service (USPHS) and the US Centers for Disease Control and Prevention (CDC) [16] as a format for delivering behavioral interventions [7]. All U.S. state residents have access to QLs funded through various mechanisms of which 76% (38/50) are described as providing proactive QL counseling [17].

Despite their proven efficacy and widespread availability, significant barriers to QL use clearly exist as most state QLs currently reach only 1% to 5% of their tobacco-using population [18]. One of the potential barriers may be that all state proactive QL models, except Wisconsin [11] and New York [19], proactively initiate subsequent contact with tobacco users but require tobacco users to make the initial contact. In the current study, we incorporated a referral technique similar to the *Fax-to-Quit *program developed by the Wisconsin Center for Tobacco Research and Intervention [11]. In this model, patient information is provided to the QL and the QL initiates the first contact. In 2004, 30% of the 12,000 callers to the Wisconsin Tobacco Quit Line were enrolled through the Fax-to-Quit program. Investigators in Oregon have reported that the *Fax-to-Quit *model in the primary care setting is feasible and cost-effective [12]. In the Oregon study, the QL was able to successfully contact 59% of subjects which is similar to the completion rate observed in our study. Future research is also required to evaluate whether reactive quitline interventions offered in the ED would result in participation rates as high as we observed with a proactive intervention.

The *Fax-to-Quit *model, however, has not been formally evaluated through the ED. Despite the promising completion rate for the QL counseling intervention in this pilot, we were only able to reach 51% of study participants for any follow-up smoking assessment. Further investigation of the efficacy of the *Fax-to-Quit *model in the ED population is warranted particularly given it has been encouraged by the American College of Emergency Physicians [20]. However, follow-up in this population is problematic, and further evaluation of techniques to improve outcome data collection is needed in order for a larger randomized controlled trial to be feasible.

## Limitations

Our pilot study was limited by small sample size, low rates of follow-up, and self-reported outcomes. We enrolled a convenience sample as we were not able to approach all eligible patients and study personnel were not always available to screen patients identified by attending staff. Therefore, we may have an unrecognized selection bias. The completion rate for the proactive quitline intervention is a descriptive outcome and is compared only to previous ED studies that have demonstrated low rates of follow-up with outpatient counseling referrals.

## Conclusion

Linking a QL to tobacco use interventions in the ED may be one alternative to providing needed tobacco use counseling to ED patients without significantly increasing demands on clinician time or resources. Compliance with the QL intervention in this pilot study was encouraging, but larger studies are required to determine if this is an accurate estimate of true participation rates for the ED population. Future studies should also focus on the development of effective protocols for identifying motivated smokers and more effective mechanisms to obtain outcome measures.

## Abbreviations

ED, Emergency Department; QL, Quitline; CI, Confidence Interval; USPHS, United States Public Health Service; FTND, Fagerström Test for Nicotine Dependence; DVT, Deep Venous Thrombosis; UTI, Urinary Tract Infection; CDC, US Center for Disease Control and Prevention.

## Competing interests

The author(s) declare that they have no competing interests.

## Authors' contributions

NEES and JOE conceived the study, designed the trial, and obtained research funding. NEES supervised the conduct of the trial and data collection. NEES drafted the manuscript, and JOE contributed substantially to its revision. NEES takes responsibility for the paper as a whole. All authors read and approved the final manuscript.

## Pre-publication history

The pre-publication history for this paper can be accessed here:


